# The symphony of open-heart surgical care: A mixed-methods study about interprofessional attitudes towards family involvement

**DOI:** 10.1080/17482631.2023.2176974

**Published:** 2023-02-22

**Authors:** Anna Drakenberg, MiaLinn Arvidsson-Lindvall, Elisabeth Ericsson, Susanna Ågren, Ann-Sofie Sundqvist

**Affiliations:** a Faculty of Medicine and Health, School of Health Sciences, Örebro University, Örebro, Sweden; b Department of Cardiothoracic and Vascular Surgery, Örebro University Hospital, Örebro, Sweden; c Faculty of Medicine and Health, University Health Care Research Centre, Örebro University, Örebro, Sweden; d Department of Health, Medical and Caring Sciences, Linköping University, Linköping, Sweden; e Department of Cardiothoracic Surgery and Department of Health, Medical and Caring Sciences, Linköping University, Linköping, Sweden

**Keywords:** Attitude, cardiac surgical procedures, family, family nursing, interprofessional research

## Abstract

**Purpose:**

The overall aim of this study was to describe the attitudes towards family involvement in care held by nurses and medical doctors working in open-heart surgical care and the factors influencing these attitudes.

**Methods:**

Mixed-methods convergent parallel design. A web-based survey was completed by nurses (*n* = 267) using the Families’ Importance in Nursing Care-Nurses Attitudes (FINC-NA) instrument and two open-ended questions, generating one quantitative and one qualitative dataset. Qualitative interviews with medical doctors (*n* = 20) were conducted in parallel, generating another qualitative dataset. Data were analysed separately according to each paradigm and then merged into mixed-methods concepts. Meta-inferences of these concepts were discussed.

**Results:**

The nurses reported positive attitudes in general. The two qualitative datasets from nurses and medical doctors resulted in the identification of seven generic categories. The main mixed-methods finding was the attitude that the importance of family involvement in care depends on the situation.

**Conclusions:**

The dependence of family involvement on the situation may be due to the patient’s and family’s unique needs. If professionals’ attitudes rather than the family’s needs and preferences determine how the family is involved, care runs the risk of being unequal.

## 1 Introduction

Open-heart surgery, e.g., coronary artery bypass grafting, aortic procedures and cardiac valve replacement, is a common treatment for cardiovascular diseases (Head et al., 2017; Stephens & Whitman, 2015). Coronary artery bypass grafting is the most frequent cardiac surgical procedure in high-income countries (Head et al., 2017; McDermott & Liang, 2021), with a rate of approximately 63 per 100 000 inhabitants in the US (McDermott & Liang, 2021) and 44 of 100 000 in western Europe (Head et al., 2017). However, open-heart surgery may lead to life-threatening complications. Because of these risks, patients need to be monitored and cared for in an advanced, highly technological postoperative environment by a multiprofessional team (Stephens & Whitman, 2015). Just as a symphony requires an orchestra, open-heart surgical care requires a multiskilled team of professionals with various perspectives on patient care. A team consisting of cardiothoracic surgeons, anaesthesiologists, intensive care nurses, surgical nurses, physiotherapists, and other healthcare professionals (Stephens & Whitman, 2015) makes interprofessional collaboration essential for care quality and safety (Pomare et al., 2020; World Health Organization, 2010). Patients are often dependent on their families during the rehabilitation phase at home (Bjørnnes et al., 2019). Family involvement in care is recommended internationally (Davidson et al., 2017; Johnson & Abraham, 2012; Shajani & Snell, 2019) and has been known to improve both patient (Eskes et al., 2019; Mackie et al., 2019) and family (Bjørnnes et al., 2019; Joseph et al., 2015) outcomes.

The meaning of family involvement in care has a broad definition in this study. Family includes not only relations by bloodline or law but also emotional relationships (Benzein, Johansson, Årestedt, & Saveman, 2008). Family involvement requires family presence, information sharing, and the facilitation of family members’ participation in shared decision making and basic care activities (Olding et al., 2016). Furthermore, family involvement means that family members should be supported and have their own needs met by health care professionals (Olding et al., 2016).

Postoperative recovery may be enhanced when the family is involved in preventive patient care targeting surgical complications (Eskes et al., 2019), leading to an improvement in family satisfaction with care (Bjørnnes et al., 2019). The risks and consequences of open-heart surgery put a strain on both the patient and the patient’s family in terms of stress and anxiety (Bjørnnes et al., 2019; Joseph et al., 2015; Kemp et al., 2020; Robley et al., 2010). The family plays a key role in rehabilitation following open-heart surgery, a responsibility they are not always prepared for (Bjørnnes et al., 2019). Preoperative anxiety is associated with impaired postoperative recovery for the patient and may double all-cause mortality after open-heart surgery (Joseph et al., 2015). It has been suggested that stress and anxiety can be reduced for both family and patients when the family is involved in the care of their ill family member (Bjørnnes et al., 2019; Mackie et al., 2019). Attitudes of the health care team towards family involvement in care influence how families are treated and involved (Bell, 2013; Benzein, Johansson, Årestedt, & Saveman, 2008; Mackie et al., 2018). An attitude may be defined as a state of believing, valuing, or feeling something that predisposes an action or behaviour (Altmann, 2008). Attitudes predispose how we act, but this does not mean that we always act according to our attitudes (Altmann, 2008). Attitudes can be either conscious or unconscious and therefore cannot be measured directly and may be illuminated in our behaviour (Bakanauskas et al., 2020). Attitudes towards family involvement in care have been known to vary between care contexts and groups of health care professionals (Al Mutair et al., 2013; Barreto et al., 2022; Benzein, Johansson, Årestedt, Berg, et al., 2008; Davis et al., 2014; Dijkman et al., 2021; Jordan et al., 2014; R. Laidsaar-Powell et al., 2017; Rosland et al., 2011; Shin et al., 2017). Nurses’ attitudes towards family involvement have been explored to some extent in the context of cardiology (Gusdal et al., 2017; Luttik et al., 2017), surgical care (Blöndal et al., 2014) and a general nursing context (Benzein, Johansson, Årestedt, Berg, et al., 2008; Østergaard et al., 2020). Nurses generally hold positive attitudes towards family involvement in care, but some variations related to nurses´ personal experiences, educational level and context of workplace have been found in previous studies (Barreto et al., 2022). There are also significant differences in nurses’ attitudes between countries (Cranley et al., 2022; Shamali et al., 2022). At times, nurses’ negative attitudes may hinder family involvement, such as holding the belief that the patient comes first and family members take time away from patient care (Mackie et al., 2018). Research on medical doctors’ (MDs) attitudes towards family involvement in care has mostly focused on primary, geriatric and oncology care settings (Dijkman et al., 2021; R. C. Laidsaar-Powell et al., 2013; Rosland et al., 2011; Shin et al., 2017). Aspects of family involvement in care from the perspective of MDs in previous studies are shared decision making, communication and family presence (Dijkman et al., 2021; Jordan et al., 2014; R. C. Laidsaar-Powell et al., 2013; Shin et al., 2017). Surgeons’ interactions with families have been reported to vary (Jordan et al., 2014). For example, some always include the family in their preoperative communications while others never do (Jordan et al., 2014). Studies including both nurses’ and MDs’ attitudes towards family involvement in the care of adult patients are limited (Al Mutair et al., 2013; Davis et al., 2014; R. Laidsaar-Powell et al., 2017). When compared, nurses (R. Laidsaar-Powell et al., 2017), patients and families (Shin et al., 2017) hold more positive attitudes towards family involvement in oncology care than MDs. On the other hand, MDs tend to be more positive than nurses to family involvement in patient-safety practices in the acute care setting (Davis et al., 2014).

Because of the complexity of capturing attitudes, a mixed-methods approach was considered beneficial for the description of attitudes towards family involvement from informants from various professions. The merging of qualitative and quantitative results enables comparisons to be made, and a more complete understanding emerges than that provided by the quantitative and qualitative results alone. The overall aim was to describe the attitudes towards family involvement in care held by nurses and medical doctors working in open-heart surgical care and the factors influencing these attitudes.

The study aim was based on the following research questions.

- How do nurses and medical doctors working in open-heart surgical care describe their attitudes towards family involvement in care and the factors affecting these attitudes? (Qualitative)
- How do nurses working in open-heart surgical care rate the importance of family involvement in nursing care? (Quantitative)
- Is age, education and/or previous experiences associated with nurses’ ratings of their attitudes towards family involvement in nursing care? (Quantitative)

To what extent do these attitudes and components converge and diverge? (Mixed-methods)

## 2 Materials and methods

### 2.1 Design

**Figure 1.**
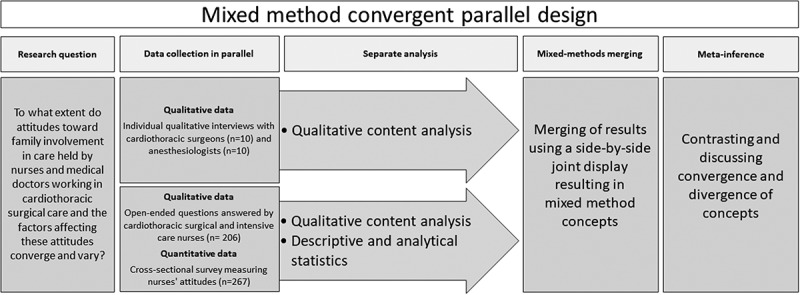
The mixed method convergent design process: Data collection, analysis, merging and meta-inference.

A mixed-methods convergent parallel design was used. This is a type of design in which qualitative and quantitative data are collected in parallel and analysed separately, and then the results are merged and meta-inferences can be made (Creswell & Plano Clark, 2018). The reporting in this study adheres to the Strengthening the Reporting of Observational Studies in Epidemiology (STROBE) guidelines (von Elm et al., 2014) and the Consolidated Criteria for Reporting Qualitative Research (COREQ) (Tong et al., 2007). Strategies to minimize validity threats in convergent mixed-method design (Creswell & Plano Clark, 2018) is applied in this study. The mixed-methods convergent parallel process is presented in Figure 1.

### 2.2 Setting

Patient autonomy and rights to participate in their own care is emphasized in c 3 § 4 of the Swedish Patient Act (SFS, 2014). In c 5 § 3, the family’s right to be involved in patient care is also established, given that the patient cannot receive information himself or herself and if it is deemed appropriate (SFS, 2014). In Sweden, open-heart surgical care is organized similarly in eight hospitals, seven of which are university hospitals. When a surgical indication is identified, the patient is referred to the cardiothoracic surgical department from the cardiology department. Patients are cared for in a surgical ward, the operating theatre, the intensive-care unit, and at some departments, a step-down unit. These units are staffed with health care professionals with various educational backgrounds, such as nurse assistants; registered nurses (some having a vocational degree and others having a bachelor’s degree); nurses with a postgraduate diploma in surgery, anaesthesiology or cardiology (some holding a master’s degree); physiotherapists (some holding a master’s degree in respiratory therapy or intensive care); occupational therapists; and MDs in training to become specialized in cardiology, cardiothoracic surgery or anaesthesiology. In addition to the abovementioned, resident cardiothoracic anaesthesiologists and surgeons work in all units. All units have close collaboration; for this study, nurses and MDs were included from the intensive care units, step-down units and/or surgical wards since these are places where family members are invited to visit. Family involvement in the operation theatre is an unusual exception in this context and foremost an issue when the patient is a child.

### 2.3 Participants

#### 2.3.1 Nurses

Nurses included in this study were recruited from all eight cardiothoracic departments in Sweden from April-November 2020. Data collection was planned for March-April 2020, but this period was the first wave of the COVID-19 pandemic in Sweden. Therefore, the departments themselves chose when to participate in this study, and they were given the opportunity to adjust the timing of their participation to their workload in relation to the strain of pandemic care. Three clinics agreed to complete the Families’ Importance in Nursing Care-Nurses Attitudes (FINC-NA) instrument from April-September 2020, while the remaining five clinics completed the instrument from September-November 2020. The nurses were instructed to answer while thinking of the normal situation when there were normal routines for family involvement in care.

The inclusion criteria were 1) being employed as a nurse at one of the wards included in the study and 2) caring for patients undergoing elective open-heart surgery and meeting these patients’ families.

After written consent was obtained from the head of each department, email addresses for the nurses were retrieved. Written information about the study was sent out via workplace email to 650 eligible participants. One week after the information was distributed, an electronic questionnaire was sent out via a unique link to each participant. Nurses willing to participate gave their consent by answering and submitting the questionnaire anonymously. Three reminders were sent to the first group, and four reminders were sent to the second group.

#### 2.3.2 Medical doctors

The MDs included in this study were recruited from three out of the seven cardiothoracic departments situated at university hospitals in Sweden. The MDs were working at the aforementioned university hospitals from April-November 2020.

The inclusion criteria were 1) being employed as an MD at one of the three cardiothoracic centres included in this part of the study and 2) treating patients undergoing elective open-heart surgery and meeting these patients’ families.

Recruitment of participants for the individual interviews started with an oral presentation in two out of three study sites. Due to travel restrictions during spring 2020, it was not possible to visit the third study site as planned to provide group information to the MDs. Written information about the study was sent via workplace email to all cardiothoracic surgeons and anaesthesiologists working at the three study sites (*n* = 133). MDs were recruited via convenience sampling, and all MDs interested in participating were included except for one who was no longer employed by the department at the time for data collection. MDs interested in participating answered the email, and an interview was scheduled.

The characteristics of the nurses and MDs are presented in Table I.

**Table I.** Demographic data of the participating medical doctors and nurses.

			**Medical doctors** (n=20)	**Nurses** (n=267)
**Age in years**, mean (SD)			52.8 (12.3)	44.0 (12.0)
		Missing n (%)	-	6 (2.2%)
**Years of clinical experience** mean (SD)			26.28 (11.1)	17.6 (11.9)
		Missing n (%)	-	-
**Sex** n (%)				
		Male	14 (70.0%)	41 (15.4%)
		Female	6 (30.0%)	221 (82.8%)
		Prefer not to say		1 (.4%)
		Missing	-	4 (1.5%)
**Degree** n (%)				
		Vocational	5 (25.0%)	110 (41.2%)
		Bachelor	-	70 (26.2%)
		Master’s (60 credits)	4 (20.0%)	73 (27.3%)
		Master’s (120 credits)	-	9 (3.4%)
		Doctoral	11 (55.0%)	1 (.4%)
		Missing	-	4 (1.5%)
**Experience of being a patient** n (%)				
	Yes		13 (65.0%)	138 (51.7%)
	No		6 (30.0%)	121 (45.3%)
	Prefer not to say		1 (5.0%)	2 (.7%)
	Missing			6 (2.2%)
**Experience of being a family member** n (%)				
	Yes		15 (75.0%)	199 (74.5%)
	No		4 (20.0%)	64 (24.0%)
	Prefer not to say		1 (5.0%)	1 (.4%)
	Missing		-	3 (1.1%)
**Medical specialty** n (%)				
	Anesthesiology		10 (50.0%)	-
	Cardiothoracic surgery		10 (50.0%)	-
**Postgraduate diploma in nursing** n (%)				
	Yes		-	160 (59.9%)
	Intensive care		-	116 (43.4%)
	Surgical care		-	6 (2.2%)
	Cardiac care		-	7 (2.6%)
	Registered nurse anesthetists		-	4 (1.5%)
	Other		-	4 (1.5%)
	Missing		-	23 (9%)
	No		-	107 (40.1%)
	Prefer not to say		-	-

### 2.4 Data collection

#### 2.4.1 Families’ importance in nursing care – nurses’ attitudes (FINC-NA)

The FINC-NA was originally developed as a generic measurement of nurses’ attitudes towards the importance of families in nursing care, and it was validated in Swedish (Benzein, Johansson, Årestedt, & Saveman, 2008). It has since been refined (Saveman et al., 2011), and the refined version was used in this study. The FINC-NA consists of 26 items and uses a 5-point Likert-type scale ranging from “totally agree” to “totally disagree” (Saveman et al., 2011). The minimum possible score for the total scale is 26, and the maximum is 130. The higher the score, the more supportive nurses’ attitudes towards families in nursing care are. The FINC-NA has four subscales. Family as a resource in nursing care (Fam-RNC) assesses positive aspects of how the family influences the nurse’s work and the importance of family presence for nursing care. Family as a conversational partner (Fam-CP) concerns the nurse’s inclusive work with families when planning care, mapping out who belongs to the family, and communicating and inviting the family to participate in care planning and nursing care. Family as a burden (Fam-B) covers negative aspects of family involvement, such as whether the nurse is stressed by the family or does not have time for families; this scale is reverse scored. Family as own resource (Fam-OR) concerns how the nurse collaborates with and supports the family, enhancing the family’s own resources as a strategy for them to cope with the situation. The α reliability coefficients are .89 for the whole instrument and between .71 to .86 for the four subscales (Saveman et al., 2011). The original authors gave their permission to use the instrument for nurses but not for MDs since it was not developed for use in the MD population.

#### 2.4.2 Open-ended questions in the instrument

The FINC-NA was concluded with two open-ended questions added by the authors of this study. The open-ended questions for the nurses were intended to ask questions similar to those for the MDs, as parallel questions facilitate the merging of results in a mixed-methods convergent parallel design (Creswell & Plano Clark, 2018).

The two questions were as follows:

1. What does family involvement mean to you?
2. What influences your attitude towards family involvement in care?

The answers to these open-ended questions were described by the participating nurses in a total of 15,919 words, which was the dataset to be included in the qualitative analysis.

#### 2.4.3 Qualitative interviews

The key structure of the FINC-NA (Saveman et al., 2011) guided the construction of semistructured questions for the qualitative interviews with the MDs. Areas covered by the interview guide were the meaning of family involvement, family members’ role in caregiving, negative and positive aspects of family involvement and what the participant thought influenced his or her attitudes towards family involvement in care.

After conducting two pilot interviews with two MDs not eligible as study participants, the first author received feedback from the co-authors on the interview technique. The pilot interviews were not included for analysis in this study. The original opening question was “What does family involvement mean to you?” This question prompted the participants answer with a general statement regarding semantics of the phrase “family involvement” as opposed to describing their own personal attitudes. The question was therefore altered to “Think back to a situation when family were involved in the care you gave. Please describe the situation.” Prompting questions such as “How was that for you?” were also used.

Twenty MDs participated in qualitative interviews in the study. The first author conducted all interviews. They were held at the place of the participant’s choice, usually in the participant’s private office at the clinic. Seven interviews were held using a videoconferencing platform (i.e., Zoom) due to national travel restrictions during the COVID-19 pandemic. The interviews lasted 24–97 minutes, with a median of 50 minutes. All interviews were audio recorded and transcribed verbatim by a professional transcriber and formed the basis of the analysis.

### 2.5 Analysis

The analysis was conducted in four separate steps consisting of 1) statistical analysis of the FINC-NA scores, 2) qualitative content analysis of the answers to the open-ended questions, 3) qualitative content analysis of the transcribed interview data and 4) mixed-methods merging and meta-inference using a side-by-side joint display.

#### 2.5.1 Statistical analysis

The quantitative data were analysed using descriptive statistics. Continuous variables are presented as the median and interquartile range (*q*1; *q*3), and categorical variables are presented as numbers and percentages. The Fam-B scale scores were reversed prior to analysis, making all scores indicate attitudes in the same direction from 1 (negative) to 5 (positive). The Mann—Whitney U test was performed for comparisons between groups by sex, educational degree, possession of a postgraduate diploma in nursing, and experience of being a patient or a family member of a patient. The correlations between age of the nurses and subscales and between years of experience as a nurse and subscales were analysed with Spearman’s rank correlation (r_s_). Missing data (1.2%) were not imputed, and cases with missing data were listwise deleted. To be included in the analysis, a minimum of 60% of the items on the FINC-NA had to be answered. The participants’ academic degrees were dichotomized into basic (vocational and bachelor’s degrees) and advanced (master’s and doctoral degrees) levels. The “prefer not to say” answer to demographic questions was treated as missing in the dichotomous group analysis. All tests were two-sided and conducted at the 5% significance level. Statistical analyses were performed using IBM SPSS Statistics (Version 26) software.

#### 2.5.2 Qualitative content analysis

All qualitative data were analysed independently by all authors who had diverse preunderstandings. The authors’ clinical backgrounds were in the cardiothoracic care setting as nurses (AD, ASS, SÅ), in general surgical care as a nurse anaesthetist (EE) and in stroke and primary care as a physiotherapist (MLAL). All authors had experience conducting qualitative analysis. Analysis of free text answers in the questionnaire and the transcribed interviews was made separately and followed Elo and Kyngäs’s (2008) description of inductive qualitative content analysis. The first author independently analysed all interviews and all the material from the open-ended questions. All data from the interviews and all the material from the open-ended questions were divided between the other co-authors for individual analysis. Initially, all authors independently analysed the material by open coding, transferring the codes to a coding sheet, and thereafter grouping codes into preliminary categories. After this, the authors’ individual analysis was compared and discussed within the research group until agreement was reached. Hence, all data were analysed individually by two authors until the grouping stage of Elo and Kyngäs’s (2008) analysis process. After discussion, agreement regarding the grouping was reached. At this stage, the first author expanded the analysis through Elo and Kyngäs’s (2008) categorization. The expanded analysis was then discussed within the research group until agreement was reached.

#### 2.5.3 Mixed-methods merging and meta-inference

This study’s mixed-methods research question was “To what extent do attitudes towards family involvement in care held by nurses and medical doctors working in open-heart surgical care and the factors affecting these attitudes converge and diverge?”. To answer this question, the three datasets were merged, using a side-by-side joint display (Creswell & Plano Clark, 2018). The merging of results was expected to yield greater insight into the phenomenon of attitudes towards family involvement in care held by nurses and MDs than each unit of data separately would. In the instances where merging is the integration procedure, a side-by-side joint display is recommended (Younas & Durante, 2022). The headings and textual descriptions of the generic categories and groupings from the two qualitative datasets were initially contrasted inductively by the first author by their overarching themes. Thereafter, the descriptions of the FINC-NA subscales were contrasted by the first author against the themes identified in the two qualitative datasets. The descriptions of the FINC-NA subscales and median values and results from analytical statistical tests in each subscale and on total scale level were organized according to the preliminary themes and their correspondence to one and another. When displayed for contrast and comparison, the content of the results was merged into concepts of convergent and divergent attitudes. At this stage, the merging and meta-inference was discussed within the research group until agreement was reached. The last step in the mixed-methods convergent parallel design process, the meta-inference of the concepts, is discussed in the discussion section of this article. An example from the side-by-side joint display table used for the mixed-methods merging and meta-inference is presented in Table II.

**Table II.** The mixed method merging and meta-inference of three datasets: An example from the side-by-side joint display analysis.

1. Mixed-method concepts	2. Qualitative results from interviews with medical doctors	2. Qualitative results from open-ended questions answered by registered nurses	3. Quantitative results from FINC-NA* answered by registered nurses	4. Meta-inference
Supporting, informing andimproving care	**Generic category**: The synthesized textual description of the content in Striving for the patient´s best **Grouping**: The synthesized textual description of the content in Advocating for the patient and Improving health **Quote**: *And when everything is perfect, then they motivate the patient in ways I can’t. They have the patient’s entire life as a source of information that I don’t have*. (Physician 12)	**Generic category**: The synthesized textual description of the content in Affecting the quality of care **Grouping**: The synthesized textual description of the content in Improved quality of care **Quote**: *Support and comfort for the patient, before and after surgery. Involvement in rehabilitation and usually better patient outcome. Being an extra pair of ears, listening to information. Sometimes giving information of importance. Family involvement can sometimes be more time-consuming but is, in my opinion, better for patient outcomes (Nurse 220)*	**Descriptions of sub-scales**: Family as a resource in nursing care (Fam-RNC) assesses positive aspects of how the family influences the nurse’s work and the importance of family presence for nursing care. Family as a conversational partner (Fam-CP) concerns the nurse’s inclusive work with families when planning care, mapping out who belongs to the family, and communicating and inviting the family to participate in care planning and nursing care. **Number of items in the FINC-NA belonging to the concept**† **Supporting, informing and improving care**: 18 out of 26 items **Medians on item level**: The items with the highest median scores (5) belong in this theme, variation between 3–5 among these 18 items.	**Convergent** Positive attitudes were convergent between paradigm and interprofessionally, nurses’ and medical doctors’ attitudes are foremost positive to family involvement in open-heart surgical care

Note: FINC-NA Families’ Importance in Nursing Care—Nurses’ Attitudes questionnaire ^†^The authors decided which items belonged to which concept after a discussion about how the sub-scale descriptions corresponded to the content of the preliminary themes developed from merging results from the two qualitative datasets.

### 2.6 Ethical considerations

The study was approved by the Swedish Ethical Review Authority (No 2019–06315), and it conforms with the principles outlined in the Declaration of Helsinki (World Medical Association, 2013). Some of the participants were recruited from the first, fourth and last authors’ workplaces. The first author conducted all interviews. The written information was at times followed up with oral information by the first or fourth author during clinical duty. This oral information was given on the request of the MD without pressure to participate. No author, except for the first author, analysed the interviews of MDs with whom they had a professional relationship. Since the data from the nurses were anonymized, professional relationships between participants and authors could not be determined in this dataset. The timing of this study could be considered burdensome for the participants, considering the workload during the first year of the COVID-19 pandemic. On the other hand, participants might have felt good about the opportunity to reflect and share their thoughts on this issue during a time when several aspects of family involvement were limited.

## 3 Results

### 3.1 Results from the FINC-NA completed by nurses

In total, 267 out of the 650 eligible nurses returned the FINC-NA with at least a 60% completion grade, giving a total response rate of 41%. Response rates from the eight clinics varied between 27.9–55.6%. Complete responses (i.e., responses on all 26 items) were returned by 222 out of the 267 included nurses. The median score for the FINC-NA total scale was 93 (Q1 = 85 Q3 = 104). Descriptive results from the total FINC-NA and the four subscales and items are presented in Table III.

**Table III.** Results for the families’ importance in nursing care – nurses’ attitudes questionnaire; item scores for the total population.

	Number (Missing)	Totally disagree/1	2	3	4	Totally agree/5	Median (Q1/Q3)	Possible range
**Families’ importance in Nursing Care- Nurses’ Attitudes (FINC-NA) total score†**	222 (45)	n (%)	n (%)	n (%)	n (%)	n (%)	93 (85/104)	26–130
**Family as a resource in nursing care (Fam-RNC)**	250 (17)						38 (34/43)	10–50
3) A good relationship with family members gives me job satisfaction	266 (1)	2 (.8%))	4 (1.5%)	33 (12.4%)	86 (32.3%)	141 (53.0%)	5.0 (4/5)	
4) Family members should be invited to take an active part in the patient’s care	266 (1)	10 (3.8%)	36 (13.5%)	87 (32.7%)	82 (30.8%)	51 (19.2%)	3.5 (3/4)	
5) The presence of family members is important to me as a nurse	267 (0)	3 (1.1%)	31 (11.6%)	65 (24.3%)	99 (37.1%)	69 (25.8%)	4.0 (3/5)	
7) The presence of family members gives me a feeling of security	265 (2)	20 (7.5%)	50 (18.9%)	111 (41.9%)	63 (23.8%)	21 (7.9%)	3.0 (2/4)	
10) The presence of family members eases my workload	266 (1)	13 (4.9%)	46 (17.3%)	118 (44.4%)	75 (28.2%)	14 (5.3%)	3.0 (3/4)	
11) Family members should be invited to actively take part in the planning of patient care	265 (2)	3 (1.1%)	13 (4.9%)	57 (21.5%)	106 (40.0%)	86 (32.5%)	4.0 (3/5)	
13) The presence of family members is important for the family members themselves	264 (3)	0 (0%)	2 (.7%)	34 (12.7%)	95 (36.0%)	133 (50.4%)	5.0 (4/5)	
20) Getting involved with families gives me a feeling of being useful	259 (8)	8 (3.1%)	28 (10.8%)	85 (32.8%)	82 (31.7%)	56 (21.6%)	4.0 (3/4)	
21) I gain a lot of worthwhile knowledge from families that I can use in my work	264 (3)	8 (3.0%)	17 (6.4%)	49 (18.6%)	107 (40.5%)	83 (31.4%)	4.0 (3/5)	
22) It is important to spend time with families	264 (3)	2 (.8%)	6 (2.3%)	43 (16.3%)	93 (35.2%)	120 (45.5%)	4.0 (4/5)	
**Family as a conversational partner (Fam-CP)**	243 (24)						28 (24/32)	8–40
1) It is important to find out who belongs to the patient’s family	264 (3)	0 (0%)	4 (1.5%)	34 (12.9%)	73 (27.7%)	153 (58.0%)	5.0 (4/5)	
6) I ask family members to take part in discussions when a patient first comes into my care	265 (2)	33 (12.5%)	33 (12.5%)	59 (22.3%)	79 (29.8%)	61 (23.0%)	4.0 (2.5/4)	
9) Discussion with family members when a patient first comes into my care saves time in my future work	265 (2)	16 (6.0%)	20 (7.5%)	82 (30.9%)	85 (32.1%)	62 (23.4%)	4.0 (3/4)	
12) I always find out who belongs to the patient’s family	265 (2)	9 (3.4%)	43 (16.2%)	62 (23.4%)	81 (30.6%)	70 (26.4%)	4.0 (3/5)	
14) I invite family members for a discussion at the end of the care period	266 (1)	47 (17.7%)	70 (26.3%)	79 (29.7%)	50 (18.8%)	20 (7.5%)	3.0 (2/4)	
15) I invite family members to take an active part in the patient´s care	262 (5)	48 (18.3%)	80 (30.5%)	89 (34.0%)	38 (14.5%)	7 (2.7%)	3.0 (2/3)	
19) I invite family members for discussions when the patient’s condition changes/deteriorates	263 (4)	5 (1.9%)	11 (4.2%)	43 (16.3%)	94 (35.7%)	110 (41.8%)	4.0 (4/5)	
24) I invite family members for discussions when planning care	259 (8)	14 (5.4%)	29 (11.2%)	69 (26.6%)	71 (27.4%)	76 (29.3%)	4.0 (3/5)	
**Family as own resource (Fam-OR)**	255 (12)						13 (11/16)	4–20
16) I ask families how I can support them	265 (2)	14 (5.3%)	49 (18.5%)	84 (31.7%)	73 (27.5%)	45 (17.0%)	3.0 (3/4)	
17) I encourage families to use their own resources so that they can cope with their situation as far as possible	263 (4)	15 (5.7%)	50 (19.0%)	87 (33.1%)	84 (31.9%)	27 (10.3%)	3.0 (3/4)	
18) I consider family members as cooperating partners	264 (3)	15 (5.7%)	36 (13.6%)	74 (28.0%)	89 (33.7%)	50 (18.9%)	4.0 (3/4)	
25) I see myself as a resource for families so that they can cope as well as possible with their situation	259 (8)	9 (3.5%)	44 (17.0%)	83 (32.0%)	92 (35.5%)	31 (12.0%)	3.0 (3/4)	
	Number (Missing)	Totally agree/1	2	3	4	Totally disagree/5	Median (Q1/Q3)	Possible range
**Family as a burden (Fam-B) reversed score**‡	254 (13)						15 (12/17)	4–20
2) The presence of family members holds me back in my work	266 (1)	2 (.7%)	23 (8.6%)	72 (27.1%)	104 (39.1%)	65 (24.4%)	4 (3/4)	
8) I do not have time to take care of families	262 (5)	8 (3.1%)	48 (18.3%)	68 (26.0%)	82 (31.3%)	56 (21.4%)	4 (3/4)	
23) The presence of family members makes me feel that they are checking up on me	262 (5)	8 (3.1%)	40 (15.3%)	78 (29.8%)	73 (27.9%)	63 (24.0%)	4 (3/4)	
26) The presence of family members makes me feel stressed	261 (6)	3 (1.1%)	30 (11.5%)	58 (22.2%)	84 (32.2%)	86 (33.0%)	4 (3/5)	

Note: ^†^Displaying the original items in the instrument Families Importance in Nursing Care- Nurses Attitudes. Published by Saveman et al. (2011) in Refinement and psychometric re-evaluation of the instrument Refinement and Psychometric Re-evaluation of the Instrument: Families’ Importance in Nursing Care—Nurses’ Attitudes. Journal of Family Nursing, 17(3), 312–329. https://doi.org/10.1177/1074840711415074. ‡The Fam-B scale was reversed prior to analysis, making ratings on all items indicate attitudes in the same direction, i.e., high score= positive attitude and low score =negative attitude; Q1=First quartile Q3=Third quartile

Internal consistency for subscales was reliable, with Cronbach’s alphas between .71 and .88 for the four subscales and .90 for the total scale. Overall FINC-NA scores were high, indicating positive attitudes towards the importance of families in nursing care. There was no significant difference in the FINC-NA total score by age, sex, years of experience as a nurse, academic level, or experience of either being a patient or being a family member to a patient. Female participants held more positive attitudes on the Fam-RNC subscale than male participants (*p* = 0.004). Nurses with an advanced educational degree (i.e., master’s or doctoral degrees) showed a significantly higher ranking on the reversed score Fam-B subscale than nurses with vocational or bachelor’s degrees (*p* = 0.003). Having a postgraduate diploma in nursing was associated with a more positive attitude towards families on the Fam-CP (*p* = 0.040) and Fam-B (*p* = 0.013) subscales.

A poor correlation to no correlation was found between the nurses’ ages and the subscales (r_s_= −.12–.08) and between years of experience as a nurse and the subscales (r_s_= −.05–.16).

Analyses of differences on the subscales and background variables are presented in Table IV.

**Table IV.** Group differences in the Families’ Importance in Nursing Care- Nurses’ Attitudes (FINC-NA), scale and subscale results.

*High score = positive attitudes on all items and scales*		Families’ Importance in Nursing Care (FINC-NA) total score possible range of 26–130				Family as a resource in nursing care (Fam-RNC) possible range of 10–50				Family as a conversational partner (Fam-CP) possible range of 8–40				Family as own resource (Fam-OR) possible range of 4–20				Family as a burden (Fam-B) *reversed score* possible range of 4–20			
		n	Median	Q1/Q3	p^†^	n	Median	Q1/Q3	p^†^	n	Median	Q1/Q3	p^†^	n	Median	Q1/Q3	p^†^	n	Median	Q1/Q3	p^†^
Sex	Female	185	93	86/105	.362	207	39	35/43	.004*	201	28	24/32	.197	212	28	24/32	.257	211	15	12/17	.131
	Male	34	93	83/98		38	35	31/39		38	27	23/31		39	27	23/31		39	16	12/18	
Experience of being a patient	Yes	110	93	85/105	.683	128	37	33/43	.275	121	28	24/32	.809	130	13	12/16	.225	130	15	12/17	.834
	No	106	93	86/104		115	39	35/43		116	28	24/31		119	13	11/15		117	15	13/17	
Experience of being family	Yes	163	93	85/104	.923	185	37	33/43	.284	179	28	25/32	.489	190	13	11/15	.875	191	15	12/17	.062
	No	55	96	85/104		61	39	35/44		60	28	23/32		61	14	11/16		59	14	12/16	
Post graduate diploma	Yes	134	94	87/104	.225	152	38	34/43	.951	146	29	25/32	.040*	152	14	12/16	.052	154	15	13/18	.013*
	No	88	92	83/104		98	38	33/43		97	27	24/30		103	13	11/16		100	14	12/16	
Degree^‡^	Basic	147	93	84/104	.413	167	38	33/43	.546	163	28	24/32	.810	171	13	11/15	.150	171	14	12/17	.003*
	Advanced	73	94	87/105		80	38	35/43		77	28	24/32		80	14	12/16		79	16	14/17	

Note: Q1=First quartile Q3=Third quartile ^†^Mann—Whitney U test *p < 0.05 ‡Degree//Basic degree= Vocational, Bachelor Advanced degree=Master, Doctoral.

### 3.2 Results from open-ended questions answered by nurses

Out of the 267 nurses included in the quantitative analysis, 206 answered at least one of the two open-ended questions. Analysis of this material generated three generic categories: *affecting the quality of care*, *including family in their mission*, and *influential aspects*. Quotes illustrating the categories are displayed in Table V.

**Table V.** The content analysis process of nurses’ attitudes towards family involvement in open-heart surgical care.

	DATASET: OPEN-ENDED QUESTIONS, NURSES			
	Quote	Open coding	Grouping	Categorization *(final generic category)*
DATASET: OPEN-ENDED QUESTIONS, NURSES				
	*They can promote health and contribute to recovery of the soul in a way I never could because our relationship is built on illness/the operation. They have a relationship reaching beyond the time when the patient is under my care. They experience the time of illness together, and that’s why it is so important that the family isn’t just distant visitors*. (Nurse 70)	Recovery of the soul	Improved quality of care	**AFFECTING THE QUALITY OF CARE**
	*It can be really wearing when a family member has a negative impact on the patient, for example, when they encourage the patient to be still in bed when the patient really needs to be ambulated or when they feed a patient who needs to practice eating independently. They help “too much”, hindering the patient´s recovery*. (Nurse 178)	Hindering postoperative recovery	Impaired quality of care	
	*I have mostly had good experiences with family members who cheered up, activated and, if necessary, helped to reorient patients who have been confused, etc. But I have also been with family members who stressed, tired out patients and inhibited reactivation. No one is cast in the same shape, and sometimes the family members help and sometimes not* (Nurse 149)	Contributes to nursing and recovery	Improved and Impaired quality of care	
		Inhibits postoperative recovery		
	*I consider myself a resource, guiding them in (probably) the worst time of their lives, making sure that they know that we are doing everything we can for their relative who is ill. It is unquestionably important to give my time and effort to the family*. (Nurse 194)	Important to attend to the family´s needs	Family health and well-being	**INCLUDING THE FAMILY IN THEIR MISSION**
	*That they are informed about the planning and delivery of care. That they are given the opportunity to share information and opinions related to the care. That they are given the opportunity to participate in care if they wish*. (Nurse 246)	The family should be given the opportunity to be involved	Promoting family involvement	
	*The culture of the workplace, my own relationship to my family and if there is time to invite the family to participate in care* (Nurse 166)	Workplace culture, time	Organizational aspects and Experiences	**INFLUENTIAL ASPECTS**
		Personal experiences		
	*I can see a change in my attitude due to my experience of being an intensive care nurse specialist. As an ICU-nurse you spend a lot of time bedside; at first, I experienced visitation by family as stressful. I was insecure. But now I really think it enriches the care. For everyone involved!* (Nurse 206)	Professional experience	Experiences	
	*If the family supports and helps one an another, or if it is more of a burden for the patient to have the family involved, the patients feeling towards his/her family has an impact on my attitude (Nurse 217)*	The family´s influence on the patient affects attitude	Family function	
	*My attitude towards family involvement is influenced by the contact I get with the family, how they behave in the patient room together with the patient. If I get a good contact with the family quickly, I ʺtake inʺ the family faster and then more easily include them in the care. If, on the other hand, there are some kind of obstacles in our communication (aggression, language barriers, attitude problems, for example) then it takes longer to establish a contact with them. (Nurse 268)*	The family’s attitude, acting and communication related to the nurse	The nurse-family relationship	

#### 3.2.1 Affecting the quality of care

The nurses believed that family involvement affected the quality of care, both in a positive and negative way. Information about the patient provided by family members was considered a means for the nurses to give personalized care and for the family to act as the patient’s voice when they could not speak for themselves. Some reported that family involvement eased the nurse’s workload and facilitated nurse—patient communication. Family members were believed to improve recovery after surgery by providing emotional and physical support for the patient. Family involvement was seen as providing the nurse a sense of security, which in turn improved care quality. If family members transferred their own stress to the patient and limited self-care activities, family involvement could be seen as hindering the patient’s recovery. The patient is the nurse´s number one priority, and nursing may become more complicated if the family consumes the nurse’s time, space, and energy. Some nurses believed that family involvement could become an intrusion on patient integrity and autonomy, an impairment of nurse—patient communication, and a safety hazard when participating in care in the intensive care unit.

#### 3.2.2 Including the family in their mission

Nurses reported that they believed that including the family in their mission was of importance for the sake of the family. Family involvement was seen as improving the family’s health, increasing the family’s sense of coherence, giving the family a sense of security, and enhancing the family´s comprehension of the course of the disease. The nurses considered themselves to have an extended responsibility to care for not only the patient but also the family. Welcoming the family, appreciating the family, and supporting the family members’ own choices regarding their level of involvement was said to be a part of the nurse’s work.

#### 3.2.3 Influential aspects

The most prominent components influencing nurses’ attitudes were their professional experiences of meeting family members as a nurse and at times their personal experiences of being a family member. Several nurses reported using their own personal preferences regarding family involvement as a guide when caring for families. The patient’s wishes and how the nurse understood the relationship and functioning within the family were also considered to affect nurses’ attitudes towards family involvement in individual situations. The nurse-family relationship was seen as influencing collaboration between the nurse and the family. Organizational conditions, such as the context, policy of care, and attitudes of colleagues, were more general components described. Family members of a person cared for in the intensive care unit for an extended period of time were often described as having a higher priority than the family of a patient who had an elective surgery without complications.

### 3.3 Results from interviews with the medical doctors

Analysis of the twenty interviews with the MDs generated four generic categories: *caring relationship*, *complicating care*, *striving for the patient’s best* and *frames of reasoning*. Quotes illustrating the categories are displayed in Table VI.

**Table VI.** The content analysis process of medical doctors’ (MDs) attitudes towards family involvement in open-heart surgical care.

	DATASET: SEMISTRUCTURED INTERVIEWS, MEDICAL DOCTORS			
	Quote	Open coding	Grouping	Categorization *(final generic category)*
DATASET: SEMISTRUCTURED INTERVIEWS, MEDICAL DOCTORS				
	*Yes, but sometimes the patient does not make it, despite all our efforts, and then it is, but the family will live on with a lot of questions and speculations about what really happened, if the relative was well taken care of. I believe it is much easier to handle grief if you have been kept well informed continuously and been well taken care of*. (MD 16)	Being well-informed eases the family’s suffering	Creating trust through information	**CARING RELATIONSHIP**
	*So I´ll stay with the one who is afraid or does not want to. And explain to her or him what it is, and I have done this for everyone else, but I´ll explain it again. What they’ll see when they get in there. (MD 2)*	Supporting the family	Attention to the relatives	
	*Then it’s almost better to isolate the patient because everything that can go wrong is highlighted by the family members, picturing worst case scenario and planning the funeral of someone far from needing one. Their anxiety is contagious, infecting the patient*. (MD 12)	Protecting the patient from the family	Acting in difficult situations	**COMPLICATING CARE**
	*The biggest disadvantage is their anxiety, anxiety and fear, and they do not calm the operated patient; it is a problem. They’re too worried and stuff. (MD 1)*	Contagious anxiety	Challenging elements	
	*Even if they are not physically present, only the knowledge that the wife is waiting at home makes them recover. That’s how it is. (MD 9)*	Meaningful involvement at a distance	Improved health for the patient	**STRIVING FOR THE PATIENT’S BEST**
	*I: And when there are no family members, what do you do then?* *P: Well, then you must … well, then the team must have a discussion about the bigger issues. And then the care team plays a greater part in listening to the patient’s wishes, collecting information about the best fit for the patient. There is something incredibly sad about that*. (MD 5)	The patient without family support	Advocating for the patient	
	*Well, I would say it depends to a great extent on what kind of care. They have, in a cardiothoracic department, not a big role in the actual care except the few, a small percentage I think, really, among the ones passing through, that have an extended hospital stay. In those cases you can really benefit from involving family members*. (MD 4)	Family involvement vary, depending on care context	The structure of care	**FRAMES OF REASONING**
	*But if you have a distanced relationship that will be, well that will persist in the care setting, I don’t think the relationships within the family are all that effected, the pre-existing type of relationship they have, when the patient is hospitalized. That will be the same (Physician 8)*	The pre-existing family relationship affects family involvement	The family´s needs	
	*Well, my thoughts are not that different regarding what I want to do, on the other hand, being new is difficult because you don’t have the experience backing up your arguments (Physician 5)*	Experience facilitates communication with families	The physician´s experiences	
	*The family’s’ involvement is also, well it is, it is in my opinion an ethical issue really, and then I’m thinking of autonomy and the principle of autonomy and so on; it is really that, that’s guiding in this case. But that is a bit of a short cut, like it always is with ethical considerations; there are, like, other aspects as well. But I think these aspects are dominating*. (MD 15)	Family involvement is an ethical issue	Ethical approach	

#### 3.3.1 Caring relationship

In the category caring relationship, the MDs described trust as being of great importance for the family and the MD. The MDs aimed for the MD-family relationship to be supportive for the family. The relationship is built on trust, providing the family with honest and situational information. The MDs balanced information regarding patient outcomes, thereby trying to prepare the family for the worst while simultaneously giving them hope and avoiding unnecessary stress. Surgeons as well as anaesthesiologists emphasized the importance of the postoperative phone call undertaken by the surgeon to the family in establishing contact between the MD and the family. Family members were seen as collaborative partners who should be welcomed, supported, and cared for. The MDs described themselves as being attentive to the strain that involvement can place on the family and explained how they encouraged family members to preserve their resources by limiting the family’s bedside presence. Supportive conversations and displays of empathy and understanding for family members were examples of acts of caring for the family.

#### 3.3.2 Complicating care

The category complicating care entails descriptions of situations when MDs perceived family involvement as challenging and examples of how the MDs acted when family involvement complicated a situation. Cultural differences regarding expectations of health care services and communication were described as demanding. Large families were considered to take space and time and were sometimes burdensome for the MDs. Difficult conversations with families regarding adverse events or death were reported as a heavy responsibility for the MDs. Worry, guilt, and fear of being accused of mistreatment were prominent feelings in connection to difficult conversations with family members expressed by some MDs. A few MDs expressed rare instances when family members could be dangerous for the patient, for example, in domestic abusive relationships. The MDs considered themselves obligated to act when problems with family involvement occurred. Restriction of visitation and shifting focus back to the patient were other actions undertaken by the MDs when problems with family involvement occurred.

#### 3.3.3 Striving for the patient’s best

The MDs noted that the family usually was striving for the patient’s best by safeguarding the patient’s interests and giving the MD information regarding the patient’s unique situation. Attitudes regarding family members acting as experts on the patient’s needs and wishes were described on a continuum from negative to positive. The family was considered to know the person who was now a patient, thereby possessing knowledge that could improve the patient’s care and recovery. Family members acting as experts on the patient’s wishes were believed by some MDs to hinder the patient from speaking for themselves. Without family support, safeguarding of the patient’s autonomy was considered to become the responsibility of the MDs and the care team. The MDs exemplified how family members could improve patient health and promote patient recovery by being physically, psychologically, and cognitively supportive. The family was viewed as giving the patient healing care and love and motivating the patient to come home.

#### 3.3.4 Frames of reasoning

The MDs’ frames of reasoning regarding their attitudes towards family involvement in care were set by their experiences, the health-care mandate, the family´s individual needs and the MD’s ethical approach. They did not believe their attitudes had changed over time, but their professional experience had made encounters with family members easier. Some MDs described how personal experiences of being a patient or being a family member had made their attitudes regarding family involvement more positive. Professional experiences of being questioned or reported for clinical errors had in some instances led to more negative attitudes. Policy and praxis regarding health care professionals’ responsibility for the family and the family’s responsibility to be involved, as well as hospital environment and time limitations, were reported to influence MD attitudes.

Some MDs stated that their attitudes towards family involvement varied between care settings. Patients experiencing complications or requiring intensive care were regarded as being in greater need of family involvement than patients undergoing elective heart surgery without complications. Some MDs thought that a person’s attitudes towards family involvement could not be generalized. Descriptions of how families should be involved in an individualized manner were highlighted. The MDs’ perceptions of the family´s experiences and relationships affected the MDs’ beliefs about the individual family’s practice of involvement. Family involvement was considered an ethical aspect of care and a duty for the MDs. A principle of treating others the same way you want to be treated yourself was commonly applied by the MDs in relation to family involvement in care.

### 3.4 Mixed-methods merged concepts

The integration involved merging the results from the quantitative and qualitative data so that a comparison could be made, and a more complete understanding emerged than provided by the qualitative or the quantitative results alone.

Preliminary themes were initially identified in the qualitative material as 1) positive attitudes, 2) negative attitudes, 3) family care, 4) components affecting attitudes. Thereafter, the descriptions of the FINC-NA subscales, the descriptive and analytical statistics and number of items corresponding to the themes was contrasted against all categories over the two qualitative datasets.

**Figure 2.**
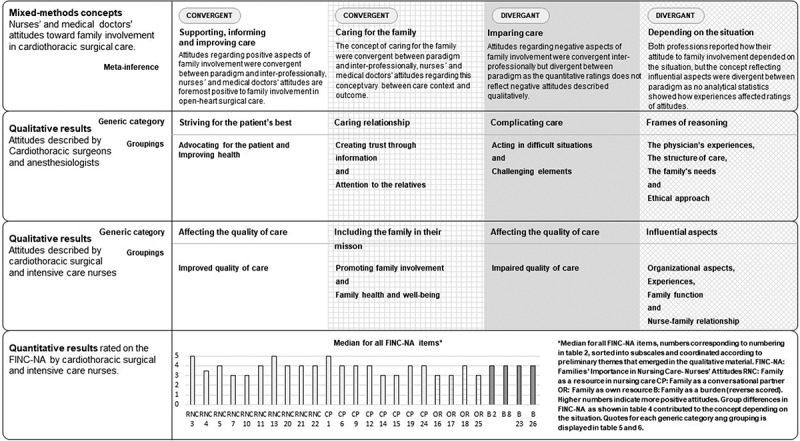
Visual joint display of qualitative, quantitative and mixed-methods results and meta-inference.

Attitudes from the three datasets were merged into four final concepts, illustrated in a visual side-by-side joint display (Figure 2). Convergent concepts were *supporting, informing and improving care* and *caring for the family*. The concepts *depending on the situation* and *impairing care* were divergent. The main finding, a concept overarching all concepts, was the attitude that the importance of family involvement in open-heart surgical care depends on the situation.

## 4 Discussion

In this study, it was found that family involvement was foremost regarded as important for the patients´ health and recovery. These attitudes were merged into the convergent concept of supporting, informing and improving care. Nurses and MDs working in open-heart surgical care considered family involvement to be important for the family members as well as for the patient under certain conditions, as shown in the convergent concept of caring for the family. Components that the participants themselves reported as affecting their attitudes were presented. These self-reported components were not always supported by the statistical analyses of the quantitative material, and they are discussed in the divergent concept depending on the situation. The negative attitudes held by nurses and MDs were not as prominent as their positive attitudes. Nevertheless, these negative attitudes need to be addressed since these areas are of greatest importance for improvement. These aspects were merged into the divergent concept impairing care.

### 4.1 Convergent concepts: supporting, informing, and improving and caring for the family

The most prominent aspect of nurses’ and MDs’ attitudes towards family involvement in open-heart surgical care was illuminated in the convergent concept *supporting, informing and improving care*. This concept entails how family involvement was seen as supporting the patient, providing valuable information for the nurses and MDs and improving quality of care and postoperative recovery. Several of these aspects have previously been highlighted by patients and family members (Bélanger et al., 2018; Mackie et al., 2019) and surgeons (Jordan et al., 2014). The predominant positive attitude towards family involvement may also be reflected in the construct of the FINC-NA. In our mixed-methods merging, 18 out of 26 items seemed to “belong” to the concept supporting, informing, and improving reflecting positive attitudes. After the refinement of the FINC-NA, ceiling effects persisted (Saveman et al., 2011). These were suggested by the original author to be explained by the socially undesirable attitude of discarding the importance of family involvement in care (Saveman et al., 2011).

The overall positive rating of attitudes in our study was supported by the qualitative material. One could, however, question the MDs’ emphasis on informing the family and question whether these practices truly are inclusive and an expression of mutual information sharing as perceived by patients and family members. MDs are often aware of how the timing and delivery of information can affect how the receiver retains the information given (Jordan et al., 2014). Family members express how shock, anger (Robley et al., 2010) and information that is unadjusted for the person´s level of health literacy (Mackie et al., 2019) may interfere with the message. Patients and family members have expressed a need for a dedicated professional who can bridge the gap in communication between the family and the care team, as well as between different members within the care team (Mackie et al., 2019).

Family, as a part of the health care mandate, was illuminated in the concept caring for the family. This concept was convergent over all datasets. It was considered to be important to care for family members in addition to the patient. *S*urgeons, in a previous study, highlighted how they consider communication to alleviate anxiety for family members (Jordan et al., 2014). The concept of caring for the family appeared to be prioritized more when the patient’s hospital stay was prolonged due to the severity of illness or complications. This attitude emerged in the qualitative datasets and could explain why the ratings on the Fam-OR scale were lower than those on the other subscales, being predominantly neutral with a median score of three for three out of four items. The Fam-OR scale concerns the importance of support for the family. The interpretation of Fam-OR scores that was made after the mixed-methods merging—that the importance of supporting the family could depend on the severity of the patients’ condition—might not have been made without the mixed-method approach. An alternative interpretation could have been that support for families was not as important as the other aspects covered by the FINC-NA. Having a postgraduate diploma in nursing was associated with more positive attitudes in our sample. The majority of nurses holding postgraduate diplomas in this study were in intensive care. The association between positive attitudes and postgraduate diplomas could thus be an expression of the attitude that family involvement is more important in the intensive care setting than in the step-down or surgical ward setting. Data specifying whether the nurses worked in the surgical ward, step-down unit or intensive care unit were not collected; therefore, it was not possible to compare these groups. Previous research regarding how the postoperative care experience differs between patients and families of patients undergoing elective emergency surgery is inconclusive but indicates that a prolonged stay in the intensive care unit predisposes them to negative experiences (Göktas et al., 2016) and that family members have specific stress-related information needs in relation to intensive care (Joseph et al., 2015). On the other hand, the need for family involvement is expressed by patients and family members of patients undergoing elective open heart surgery regardless of complications and adverse events (Bjørnnes et al., 2019; Joseph et al., 2015; Kemp et al., 2020). The patient and family experience on this topic seems to need further investigation prior to directing resources in either course.

### 4.2 Divergent concepts: depending on the situation and impairing care

Components affecting the nurses’ and MDs’ attitudes towards family involvement were merged into the divergent concept of depending on the situation. The MDs and the nurses stated that their attitudes regarding family involvement depended on several factors, such as family functioning, organizational factors, and care setting. The ethical approach was an influential aspect reported by the MDs in this study that is important to investigate further when exploring health care professionals’ attitudes towards family involvement in care since it is not asked about in the FINC-NA. The nurses in this study did not report about the ethical aspect of family involvement in their care in the qualitative data set to the same degree as the MDs. Divergence in this aspect might be due to differences between nurses and MDs views on why and how family involvement is important.

Regarding experiences being influential on the nurses’ and MDs’ attitudes, the concept was divergent. Both nurses and MDs described how having the experience of being a family member influenced their attitude towards family involvement, but there were no statistically significant differences regarding these aspects in the quantitative material. The divergence could be explained by methodology and differences between paradigms. It could be hypothesized that the nurses and MDs participating in this study whose attitudes were affected by their experiences elaborated on this in the qualitative material. In the quantitative material, the experiences of being a patient or family member was reported as either “yes” or “no”, hence both significant and nonsignificant experiences for the persons’ attitudes were reported. For example, the experience of being an adult child to a parent with a severe chronical condition may influence ones’ attitude towards family involvement in care to a greater extent compared to being an adult child to a parent in need of hospital care on one occasion. In the quantitative dataset this was not adjusted for. Another interpretation of this divergent finding is that perhaps nurses’ and MDs’ subjective beliefs about how their personal experiences influence their attitudes cannot be generalized statistically on group level. Evidence regarding whether personal health care experiences affect the overall rating in the FINC-NA from previous studies is contradictory; some have not found this association (Blöndal et al., 2014; Østergaard et al., 2020), while others have (Linnarsson et al., 2015). During the development of the FINC-NA, it was learned that the answers could not be dichotomized because the answer depended on the situation (Benzein, Johansson, Årestedt, & Saveman, 2008), supporting the concept of depending on the situation in this study. The influence of organizational conditions was reported as divergent over the three datasets regarding having a lack of time for families. A few MDs and several nurses reported having a lack of time for families in the qualitative material. This finding was not reflected in the FINC-NA Fam-B subscale, where all items had a median of four, indicating a general attitude of having time for families in this population of nurses as one item specifically asks about this in the Fam-B subscale. Time restraints have previously been described by nurses as a factor influencing their involvement of families in care, stating that their number one priority is the patient (Mackie et al., 2018).

Negative attitudes over the three datasets were merged into the concept of impairing care. How family involvement may impair patient care and recovery was described in the two qualitative datasets, making it convergent between the nurses and MDs. The nurses and MDs also expressed that family involvement could complicate their work. This was not shown in the quantitative results in the Fam-B scale, making the concept divergent between paradigms. There are no questions on the FINC-NA asking about how family involvement can be harmful for the patient, which implies divergence between the quantitative and qualitative material. This is an attitude in need of further exploration in future research. In our study, the MDs described having some difficulties caring for families of various cultural backgrounds. This was interpreted as divergent from the nurses’ experiences, possibly due to differences in data collection strategies. Perhaps, describing negative aspects and experiences would require time for reflection and report building, which is more commonly achieved during a qualitative interview compared to answering a survey. In a meta-synthesis of nurses’ experiences of caring for culturally diverse families in hospitals (Murcia & Lopez, 2016), experiences similar to those expressed by the MDs in this study were found. Difficulties communicating, a lack of space for all family members and the violation of visitation policy are some examples of barriers described by nurses in relation to culturally diverse families (Murcia & Lopez, 2016).

In health care, it is important to provide competent transcultural care (Health Research & Educational Trust, 2013). Patient and family stressors related to open-heart surgery are contextual and have cultural dimensions (Sedaghat et al., 2019). Understanding and flexibility are key when caring for culturally diverse families (Murcia & Lopez, 2016). Transcultural competence involves an understanding and exploration of health-related beliefs in different cultures and contexts (Health Research & Educational Trust, 2013). The illumination of beliefs about illness and health is also a core concept in family-centred care that is applicable to a broad spectrum of care contexts (Shajani & Snell, 2019). Therefore, it could be that education on family-centred care practices, such as illuminating families’ health and illness beliefs, could reduce negative attitudes and facilitate family involvement in care when caring for families from different cultural backgrounds in this context.

Patients and family members have, as previously stated, expressed a need for a dedicated professional who can bridge the gap in communication between the family and the care team. To enhance family involvement in care, this bridging professional would preferably have competency in family-centred care. With a family-centred care approach, the family is treated as a system in which all parts affect one and another (Bell, 2013; Shajani & Snell, 2019). Health and illness are considered a family affair, and the most effective care strategies are those targeting the whole system at once (Bell, 2013; Shajani & Snell, 2019). Competency in family-centred care would diminish the subjective practice of having family involvement depending on the situation from the professionals’ perspective and instead having the family preferences as the main focus when planning and delivering open-heart surgical care. Family-centred care with the involvement of family cannot be accomplished if not approached interprofessionally (Naef et al., 2020). It is therefore important to have a team-based approach if family-centred care is introduced in the context of open-heart surgery.

### 4.3 Strengths and limitations

This study has strengths and limitations that need to be addressed. By using a joint display in the merging and meta-inference processes, validity of these inferences was strengthened (Creswell & Plano Clark, 2018). The interview guide used in this study contributed to consistency in qualitative data collection and therefore enhanced dependability. Furthermore, the interview guide was influenced by the FINC-NA, and the open-ended questions for the nurses were intended to ask questions similar to those for the MDs. Using parallel questions between different groups in a mixed-methods convergent parallel design facilitates the merging process and is a strategy to minimize validity threats in mixed-method convergent design studies (Creswell & Plano Clark, 2018). Having different populations in the two paradigms made the mixed-methods merging and inference in this study complicated, as previously described in the literature (Creswell & Plano Clark, 2018). On the other hand, the inclusion of different populations for the different data collection strategies facilitated the usage of appropriate quality criteria according to paradigm, important for minimizing validity threats in mixed-methods studies (Creswell & Plano Clark, 2018). The FINC-NA has previously been applied to MDs working in neonatal care, with the substitution of the term “nursing care” for “care” (Naef et al., 2020). Even though the present study shows many similarities in attitudes between nurses and MDs, there are reasons to believe that an instrument developed for nurses’ attitudes towards family involvement in nursing care should not be directly applied to MDs attitudes towards family involvement in care as the instrument is not validated for the MD population. Another potential validity threat in convergent mixed-method studies is “failing to resolve disconfirming results” (Creswell & Plano Clark, 2018, p. 251). The authors of this study consider that suggestions for resolving the inferences of divergent concepts have been presented. However, wether these inferences can be considered valid is debatable.

The multidisciplinary composition of the research group may have contributed to the strengthening of credibility regarding qualitative analysis. Credibility was further enhanced by recruiting study participants from several different departments. Site credibility reduces the risk of having local factors influence the results (Shenton, 2004). Regarding transferability and generalizability, one problem was mutual between the paradigms, that is, selection bias. The aspiration to recruit all 650 patients who fulfilled the inclusion criteria in the cross-sectional study was part of the attempt to enhance the generalizability. The low response rate, however, limited this effect. The low response rate could be explained to some extent by the high strain on nurses during the COVID-19 pandemic in 2020. It is possible that the persons willing to participate in this study were the ones most favourable towards family involvement in the open-heart surgical care setting. This could, on the other hand, also be an advantage in the qualitative paradigm, where interested participants may have been able to provide richer data (Patton, 2015). The MDs participating in this study may also have considered the contribution to research to be important since 55% of them held a doctoral degree. Interviewing via a videoconferencing platform (i.e., Zoom) could have affected the richness of the data. The richness of the data could also have been affected by the fact that the first author had a professional relationship with some of the interviewees. These MDs could have responded in a more positive way, giving more positive self-descriptions or responding in a way they believed that the first author would want them to, thereby leading to socially desirable responses (Malham & Saucier, 2016). However, the elaborative answers from the MDs and their willingness to share their experiences implied that this was not a problem in the present study. The restrictions of family visitations during the COVID-19 pandemic and the heavy workload during the data collection period could have influenced the results of this study. One strategy targeting this limitation was the instructions to both nurses and MDs to answer while having the normal situation in mind.

## 4.4 Conclusions

This study has provided knowledge regarding attitudes among nurses and MDs towards family involvement in open-heart surgical care. Foremost, positive attitudes, including views on how family involvement improves postoperative recovery, have been illuminated and could be regarded as a basis for family-centred care practices. Some areas of improvement in terms of negative attitudes could be targeted by implementing structured assessment of family functioning and illness beliefs. Competencies in family-centred practices and transcultural care could enhance family involvement in the open-heartcare context. There are organizational demands to further improve family involvement, such as prioritizing time, providing physical space for families and having family-centred policies. Family involvement in open-heart surgical care may be necessary due to the patient’s and family’s unique needs in relation to open-heart surgery. Identifying those needs, as opposed to letting one’s own unconscious personal beliefs determine the extent of family involvement, demands ethical reasoning and family care competency among all members of the team.

## Data Availability

Due to the nature of this research, participants of this study did not agree for their data to be shared publicly, so supporting data is not available.

## Author controbutions

Study conception and design was performed by AD, EE, SÅ and ASS. AD collected the data. All authors participated in data analysis. The first draft of the manuscript was written by AD, and all authors gave critical feedback and edited previous versions of the manuscript. All authors read and approved the final version of the manuscript.

## References

[cit0001] Al Mutair, A., Plummer, V., O’brien, A. P., & Clerehan, R. (2013). Attitudes of healthcare providers towards family involvement and presence in adult critical care units in Saudi Arabia: A quantitative study. *Journal of Clinical Nursing*, 23(5–6), 744–19. 10.1111/jocn.12520 24734275

[cit0002] Altmann, T. K. (2008). Attitude: A concept analysis. *Nursing Forum*, 43(3), 144–150. 10.1111/j.1744-6198.2008.00106.x 18715347

[cit0003] Bakanauskas, A. P., Kondrotienė, E., & Puksas, A. (2020). The theoretical aspects of attitude formation factors and their impact on health behaviour. *Management of Organizations: Systematic Research*, 83(1), 15–36. 10.1515/mosr-2020-0002

[cit0004] Barreto, M. S., Marquete, V. F., Camparoto, C. W., García‐vivar, C., Barbieri‐figueiredo, M. D. C., & Marcon, S. S. (2022). Factors associated with nurses’ positive attitudes towards families’ involvement in nursing care: A scoping review. *Journal of Clinical Nursing*, 31(23–24), 3338–3349. 10.1111/jocn.16226 35083808 PMC9786255

[cit0005] Bélanger, L., Desmartis, M., & Coulombe, M. (2018). Barriers and facilitators to family participation in the care of their hospitalized loved ones. *Patient Experience Journal*, 5(1), 56–65. 10.35680/2372-0247.1250

[cit0006] Bell, J. M. (2013). Family nursing is more than family centered care. *Journal of Family Nursing*, 19(4), 411–417. 10.1177/1074840713512750 24227014

[cit0007] Benzein, E., Johansson, P., Årestedt, K. F., Berg, A., & Saveman, B. I. (2008). Families’ importance in nursing care. *Journal of Family Nursing*, 14(1), 97–117. 10.1177/1074840707312716 18281645

[cit0008] Benzein, E., Johansson, P., Årestedt, K. F., & Saveman, B. -I. (2008). Nurses’ attitudes about the importance of families in nursing care. *Journal of Family Nursing*, 14(2), 162–180. 10.1177/1074840708317058 18480033

[cit0009] Bjørnnes, A. K., Moons, P., Parry, M., Halvorsen, S., Tønnessen, T., & Lie, I. (2019). Experiences of informal caregivers after cardiac surgery: A systematic integrated review of qualitative and quantitative studies. *BMJ Open*, 9(11), e032751. 10.1136/bmjopen-2019-032751 PMC6858143 31719093

[cit0010] Blöndal, K., Zoëga, S., Hafsteinsdottir, J. E., Olafsdottir, O. A., Thorvardardottir, A. B., Hafsteinsdottir, S. A., & Sveinsdóttir, H. (2014). Attitudes of registered and licensed practical nurses about the importance of families in surgical hospital units. *Journal of Family Nursing*, 20(3), 355–375. 10.1177/1074840714542875 25026965

[cit0011] Cranley, L. A., Lam, S. C., Brennenstuhl, S., Kabir, Z. N., Boström, A. M., Leung, A., & Konradsen, H. (2022). Nurses’ attitudes toward the importance of families in nursing care: A multinational comparative study. *Journal of Family Nursing*, 28(1), 69–82. 10.1177/10748407211042338 34493109 PMC8814953

[cit0012] Creswell, J. W., & Plano Clark, V. L. (2018). *Designing and conducting mixed methods research* (Third ed.). SAGE.

[cit0013] Davidson, J. E., Aslakson, R. A., Long, A. C., Puntillo, K. A., Kross, E. K., Hart, J., Cox, C. E., Wunsch, H., Wickline, M. A., Nunnally, M. E., Netzer, G., Kentish-Barnes, N., Sprung, C. L., Hartog, C. S., Coombs, M., Gerritsen, R. T., Hopkins, R. O., Franck, L. S., Skrobik, Y., Kon, A. A., and Curtis, J. R. (2017). Guidelines for family-centered care in the neonatal, pediatric, and adult ICU. *Critical Care Medicine*, 45(1), 103–128. 10.1097/ccm.0000000000002169 27984278

[cit0014] Davis, R., Savvopoulou, M., Shergill, R., Shergill, S., & Schwappach, D. (2014). Predictors of healthcare professionals’ attitudes towards family involvement in safety-relevant behaviours: A cross-sectional factorial survey study. *BMJ Open*, 4(9), e005549. 10.1136/bmjopen-2014-005549 PMC4158212 25186154

[cit0015] Dijkman, B. L., Luttik, M. L., Van der Wal-Huisman, H., Paans, W., & van Leeuwen, B. L. (2021). Factors influencing family involvement in treatment decision-making for older patients with cancer: A scoping review. *Journal of Geriatric Oncology*, 13(4), 391–397. 10.1016/j.jgo.2021.11.003 34776380

[cit0016] Elo, S., & Kyngäs, H. (2008). The qualitative content analysis process. *Journal of Advanced Nursing*, 62(1), 107–115. 10.1111/j.1365-2648.2007.04569.x 18352969

[cit0017] Eskes, A. M., Schreuder, A. M., Vermeulen, H., Nieveen van Dijkum, E. J. M., & Chaboyer, W. (2019). Developing an evidence-based and theory informed intervention to involve families in patients care after surgery: A quality improvement project. *International Journal of Nursing Sciences*, 6(4), 352–361. 10.1016/j.ijnss.2019.09.006 31728386 PMC6838870

[cit0018] Göktas, S. B., Yildiz, T., Nargiz, S. K., & Gur, O. (2016). A comparison of the intensive care experiences of emergency and elective cardiac surgery patients. *Nigerian Journal of Clinical Practice*, 19(2), 284–289. 10.4103/1119-3077.175963 26856296

[cit0019] Gusdal, A. K., Josefsson, K., Thors Adolfsson, E., & Martin, L. (2017). Nurses’ attitudes toward family importance in heart failure care. *European Journal of Cardiovascular Nursing*, 16(3), 256–266. 10.1177/1474515116687178 28051331

[cit0020] Head, S. J., Milojevic, M., Taggart, D. P., & Puskas, J. D. (2017). Current practice of state-of-the-art surgical coronary revascularization. *Circulation*, 136(14), 1331–1345. 10.1161/circulationaha.116.022572 28972063

[cit0021] Health Research & Educational Trust. (2013) . *Becoming a culturally competent health care organization*.

[cit0022] Johnson, B. H., & Abraham, M. R. (2012). *Partnering with patients, residents, and families: A resource for leaders of hospitals, ambulatory care settings, and long-term care communities*. Institute for Patient-and Family-Centered Care.

[cit0023] Jordan, A. L., Rojnica, M., Siegler, M., Angelos, P., & Langerman, A. (2014). Surgeon-family perioperative communication: Surgeons’ self-reported approaches to the “surgeon-family relationship”. *Journal of the American College of Surgeons*, 219(5), 958–967. 10.1016/j.jamcollsurg.2014.05.019 25256372

[cit0024] Joseph, H. K., Whitcomb, J., & Taylor, W. (2015). Effect of anxiety on individuals and caregivers after coronary artery bypass grafting surgery. *Dimensions of Critical Care Nursing*, 34(5), 285–288. 10.1097/dcc.0000000000000137 26244244

[cit0025] Kemp, K. A., Naqvi, F., Quan, H., Paolucci, E. O., Knudtson, M. L., & Santana, M. J. (2020). Eliciting patient experiences about their care after cardiac surgery. *CJC Open*, 3(4), 427–433. 10.1016/j.cjco.2020.11.016 34027345 PMC8129438

[cit0026] Laidsaar-Powell, R. C., Butow, P. N., Bu, S., Charles, C., Gafni, A., Lam, W. W., Jansen, J., McCaffery, K. J., Shepherd, H. L., Tattersall, M. H., & Juraskova, I. (2013). Physician-patient-companion communication and decision-making: A systematic review of triadic medical consultations. *Patient Education and Counseling*, 91(1), 3–13. 10.1016/j.pec.2012.11.007 23332193

[cit0027] Laidsaar-Powell, R., Butow, P., Bu, S., Fisher, A., & Juraskova, I. (2017). Oncologists’ and oncology nurses’ attitudes and practices towards family involvement in cancer consultations. *European Journal of Cancer Care*, 26(1), e12470. 10.1111/ecc.12470 26931469

[cit0028] Linnarsson, J. R., Benzein, E., & Årestedt, K. (2015). Nurses’ views of forensic care in emergency departments and their attitudes, and involvement of family members. *Journal of Clinical Nursing*, 24(1–2), 266–274. 10.1111/jocn.12638 24890984

[cit0029] Luttik, M., Goossens, E., Ågren, S., Jaarsma, T., Mårtensson, J., Thompson, D. R., Moons, P., & Strömberg, A. (2017). Undertaking Nursing Interventions Throughout Europe (UNITE) research group. *European Journal of Cardiovascular Nursing*, 16(4), 299–308. 10.1177/1474515116663143 27470053

[cit0030] Mackie, B. R., Marshall, A., & Mitchell, M. (2018). Acute care nurses’ views on family participation and collaboration in fundamental care. *Journal of Clinical Nursing*, 27(11–12), 2346–2359. 10.1111/jocn.14185 29171145

[cit0031] Mackie, B. R., Mitchell, M., & Marshall, A. P. (2019). Patient and family members’ perceptions of family participation in care on acute care wards. *Scandinavian Journal of Caring Sciences*, 33(2), 359–370. 10.1111/scs.12631 30507038

[cit0032] Malham, P. B., & Saucier, G. (2016). The conceptual link between social desirability and cultural normativity. *International Journal of Psychology*, 51(6), 474–480. 10.1002/ijop.12261 26914819

[cit0033] McDermott, K. W., & Liang, L. (2021). Overview of operating room procedures during inpatient stays in U.S. hospitals, 2018. Agency for Healthcare Research and Quality (US). https://www.hcup-us.ahrq.gov/reports/statbriefs/sb281-Operating-Room-Procedures-During-Hospitalization-2018.pdf 34637208

[cit0034] Murcia, S. E., & Lopez, L. (2016). The experience of nurses in care for culturally diverse families: A qualitative meta-synthesis. *Revista Latino-Americana de Enfermagem*, 24, e2718. 10.1590/1518-8345.1052.2718 27384469 PMC4964299

[cit0035] Naef, R., Kläusler-Troxler, M., Ernst, J., Huber, S., Dinten-Schmid, B., Karen, T., & Petry, H. (2020). Translating family systems care into neonatology practice: A mixed method study of practitioners’ attitudes, practice skills and implementation experience. *International Journal of Nursing Studies*, 102, 103448. 10.1016/j.ijnurstu.2019.103448 31726312

[cit0036] Olding, M., McMillan, S. E., Reeves, S., Schmitt, M. H., Puntillo, K., & Kitto, S. (2016). Patient and family involvement in adult critical and intensive care settings: A scoping review. *Health Expectations*, 19(6), 1183–1202. 10.1111/hex.12402 27878937 PMC5139045

[cit0037] Østergaard, B., Clausen, A. M., Agerskov, H., Brødsgaard, A., Dieperink, K. B., Funderskov, K. F., Nielsen, D., Sorknæs, A. D., Voltelen, B., & Konradsen, H. (2020). Nurses’ attitudes regarding the importance of families in nursing care: A cross‐sectional study. *Journal of Clinical Nursing*, 29(7–8), 1290–1301. 10.1111/jocn.15196 31971287

[cit0038] Patton, M. Q. (2015). *Qualitative research & evaluation methods: Integrating theory and practice*. SAGE Publications.

[cit0039] Pomare, C., Long, J. C., Churruca, K., Ellis, L. A., & Braithwaite, J. (2020). Interprofessional collaboration in hospitals: A critical, broad-based review of the literature. *Journal of Interprofessional Care*, 34(4), 509–519. 10.1080/13561820.2019.1702515 31928245

[cit0040] Robley, L., Ballard, N., Holtzman, D., & Cooper, W. (2010). The experience of stress for open heart surgery patients and their caregivers. *Western Journal of Nursing Research*, 32(6), 794–813. 10.1177/0193945910361469 20696847

[cit0041] Rosland, A. M., Piette, J. D., Choi, H., & Heisler, M. (2011). Family and friend participation in primary care visits of patients with diabetes or heart failure. *Medical Care*, 49(1), 37–45. 10.1097/mlr.0b013e3181f37d28 21102357 PMC3712763

[cit0042] Saveman, B. I., Benzein, E. G., Engström, Å. H., & Årestedt, K. (2011). Refinement and psychometric reevaluation of the instrument. *Journal of Family Nursing*, 17(3), 312–329. 10.1177/1074840711415074 21813813

[cit0043] Sedaghat, S., Rostami, S., Ebadi, A., & Fereidooni-Moghadam, M. (2019). Stressors in open-heart surgery patients: A qualitative study. *ARYA Atherosclerosis*, 15(4), 192–200. 10.22122/arya.v15i4.1840 31819753 PMC6884732

[cit0044] SFS 2014:821. (2014). Patientlag [Swedish patient act]. Socialdepartementet. https://www.riksdagen.se/sv/dokument-lagar/dokument/svensk-forfattningssamling/patientlag-2014821_sfs-2014-821

[cit0045] Shajani, Z., & Snell, D. (2019). *Wright & Leahey’s nurses and families: A guide to family assessment and intervention*. F.A. Davis Company.

[cit0046] Shamali, M., Esandi Larramendi, N., Østergaard, B., Barbieri-Figueiredo, M., Brødsgaard, A., Canga-Armayor, A., Dieperink, K. B., Garcia-Vivar, C., Konradsen, H., Nordtug, B., Lambert, V., Mahrer-Imhof, R., Metzing, S., Nagl-Cupal, M., Imhof, L., Svavarsdottir, E. K., Swallow, V., & Luttik, M. L. (2022). Nurses’ attitudes towards family importance in nursing care across Europe. *Journal of Clinical Nursing*. Advance online publication. 10.1111/jocn.16456 35818317

[cit0047] Shenton, A. K. (2004). Strategies for ensuring trustworthiness in qualitative research projects. *Education for Information*, 22(2), 63–75. 10.3233/efi-2004-22201

[cit0048] Shin, D. W., Cho, J., Roter, D. L., Kim, S. Y., Yang, H. K., Park, K., Kim, H. J., Shin, H. Y., Kwon, T. G., & Park, J. H. (2017). Attitudes toward family involvement in cancer treatment decision making: The perspectives of patients, family caregivers, and their oncologists. *Psycho-Oncology*, 26(6), 770–778. 10.1002/pon.4226 27437905

[cit0049] Stephens, R. S., & Whitman, G. J. R. (2015). Postoperative critical care of the adult cardiac surgical patient. Part I. *Critical Care Medicine*, 43(7), 1477–1497. 10.1097/ccm.0000000000001059 25962078

[cit0050] Tong, A., Sainsbury, P., & Craig, J. (2007). Consolidated criteria for reporting qualitative research (COREQ): A 32-item checklist for interviews and focus groups. *International Journal for Quality in Health Care*, 19(6), 349–357. 10.1093/intqhc/mzm042 17872937

[cit0051] von Elm, E., Altman, D. G., Egger, M., Pocock, S. J., Gøtzsche, P. C., & Vandenbroucke, J. P. (2014). The strengthening the reporting of observational studies in epidemiology (STROBE) statement: Guidelines for reporting observational studies. *International Journal of Surgery*, 12(12), 1495–1499. 10.1016/j.ijsu.2014.07.013 25046131

[cit0052] World Health Organization. (2010). Framework for action on interprofessional education and collaborative practice. https://www.who.int/publications/i/item/framework-for-action-on-interprofessional-education-collaborative-practice? 21174039

[cit0053] World Medical Association. (2013). World medical association declaration of helsinki: Ethical principles for medical research involving human subjects. *JAMA*, 310(20), 2191–2194. 10.1001/jama.2013.281053 24141714

[cit0054] Younas, A., & Durante, A. (2022). Decision tree for identifying pertinent integration procedures and joint displays in mixed methods research. *Journal of Advanced Nursing*, Advance online publication. 10.1111/jan.15536 36524303

